# Assessment of Autonomic Nervous System Dysfunction in the Early Phase of Infection With SARS-CoV-2 Virus

**DOI:** 10.3389/fnins.2021.640835

**Published:** 2021-06-21

**Authors:** Branislav Milovanovic, Vlado Djajic, Dragana Bajic, Aleksandra Djokovic, Tatjana Krajnovic, Sladjana Jovanovic, Antonija Verhaz, Pedja Kovacevic, Miodrag Ostojic

**Affiliations:** ^1^Neurocardiology Lab, Department of Cardiology, University Hospital Medical Center Bezanijska kosa, Belgrade, Serbia; ^2^Faculty of Medicine, University of Belgrade, Belgrade, Serbia; ^3^Neurology Clinic, University Clinical Centre of the Republic of Srpska, Banja Luka, Bosnia and Herzegovina; ^4^Faculty of Technical Sciences, University of Novi Sad, Novi Sad, Serbia; ^5^Division of Interventional Cardiology, Department of Cardiology, University Hospital Medical Center Bezanijska kosa, Belgrade, Serbia; ^6^Community Health Care Center “Dr J. J. Zmaj”, Stara Pazova, Serbia; ^7^Telekom Srbija a.d., Belgrade, Serbia; ^8^Institute for Cardiovascular Diseases “Dedinje”, Belgrade, Serbia

**Keywords:** COVID-19, autonomic nervous system, cardiovascular reflex test, heart rate variability, autonomic neuropathy

## Abstract

**Background:**

We are facing the outburst of coronavirus disease 2019 (COVID-19) defined as a serious, multisystem, disorder, including various neurological manifestations in its presentation. So far, autonomic dysfunction (AD) has not been reported in patients with COVID-19 infection.

**Aim:**

Assessment of AD in the early phase of infection with severe acute respiratory syndrome coronavirus 2 (SARS-CoV-2 virus).

**Patients and methods:**

We analyzed 116 PCR positive COVID-19 patients. After the exclusion of 41 patients with associate diseases (CADG), partitioned to patients with diabetes mellitus, hypertension, and syncope, the remaining patients were included into a severe group (45 patients with confirmed interstitial pneumonia) and mild group (30 patients). Basic cardiovascular autonomic reflex tests (CART) were performed, followed by beat-to-beat heart rate variability (HRV) and systolic and diastolic blood pressure variability (BPV) analysis, along with baroreceptor sensitivity (BRS). Non-linear analysis of HRV was provided by Poincare Plot. Results were compared to **77** sex and age-matched controls.

**Results:**

AD (sympathetic, parasympathetic, or both) in our study has been revealed in 51.5% of severe, 78.0% of mild COVID-19 patients, and the difference compared to healthy controls was significant (*p* = 0.018). Orthostatic hypotension has been established in 33.0% COVID-19 patients compared to 2.6% controls (*p* = 0.001). Most of the spectral parameters of HRV and BPV confirmed AD, most prominent in the severe COVID-19 group. BRS was significantly lower in all patients (severe, mild, CADG), indicating significant sudden cardiac death risk.

**Conclusion:**

Cardiovascular autonomic neuropathy should be taken into account in COVID-19 patients’ assessment. It can be an explanation for a variety of registered manifestations, enabling a comprehensive diagnostic approach and further treatment.

## Introduction

At the end of 2019, the world has faced the coronavirus disease 2019 (COVID-19) caused by the severe acute respiratory syndrome coronavirus 2 (SARS-CoV-2)^1^. The number of infected people is measured in tens of millions (Dong et al., 2020; World Health Organization, 2020b). Although it has initially been recognized as a serious pulmonary disease, other symptoms were soon noticed and described (Guan et al., 2020; Huang et al., 2020). Among them, cardiovascular manifestations gain noticeable entanglement (Guo et al., 2020). Pathophysiology of COVID-19 cardiovascular involvement comprehends direct cardiovascular damage due to bondage of SARS-CoV-2 to angiotensin-converting enzyme 2 (ACE2) receptor, highly expressed in cardiovascular tissues (Epelman et al., 2008; Gallagher et al., 2008; Oudit et al., 2009; Walters et al., 2017; Tsatsakis et al., 2020; Xu et al., 2020) and indirect, produced by endothelial dysfunction in the systemic inflammatory response (cytokine storm), hypercoagulability, hypoxia and consequential supply-demand mismatch (Fried et al., 2020; Zheng et al., 2020). Hence, a variety of cardiovascular manifestations has been described: from myocardial ischemia and myocardial infarction (type 1 and 2), myocarditis and arrhythmias to cardiomyopathy, heart failure, and cardiogenic shock (Gupta et al., 2020). Arrhythmias in COVID-19, primarily those leading to cardiac arrest, acknowledge special concern. Their prevalence might be attributable to metabolic abnormalities, hypoxia, and severe myocardial damage, but also the administration of drugs which leads to prolongation of QTc interval. Moreover, it is proposed that during acute infections, systemic inflammation rapidly induces cytokine-mediated ventricular electrical remodeling and significant QTc prolongation, regardless of concomitant antimicrobial therapy (Lazzerini et al., 2020). Still, the impact of SARS-CoV-2 on neuromodulatory mechanisms remains unknown.

Almost four decades ago, animal studies validated the impact of the autonomic nervous system (ANS) on the cardiac cycle as an adaptive mechanism (Koizumi et al., 1983, 1985; Manolis et al., 2020). With its two arms, sympathetic (SNS) and parasympathetic (PSNS), ANS plays a crucial role in cardiac, atrial and ventricular, arrhythmogenesis (Manolis et al., 2020). The increased attention is devoted to the various markers of autonomic activity, as methods for identifying patients at risk for sudden death (Lahiri et al., 2008). Measurement of heart rate variability (HRV) as a marker of sympathovagal balance, along with various ANS function tests, are proposed as non-invasive risk stratification models in numerous studies (No authors, 1996; Freeman and Chapleau, 2013).

Direct viral invasion of neural parenchyma or via retrograde axonal transport could be a mechanism (along with the aforementioned pro-inflammatory and pro-thrombotic state) for various neurological manifestations in COVID-19 (Koralnik and Tyler, 2020). It is reasonable to presume that the same pathophysiology pathways affect ANS, provoking different disturbances in various organ systems.

Dani et al. (2021), in their recently published rapid report, anticipated a variety of autonomic instability which will develop after the acute phase of COVID-19. Although several papers deal with the mechanisms of neuromodulation in patients with COVID-19, functional analysis of the cardiac ANS in the early stages of COVID infections is lacking.

The objective of our study was to assess ANS dysfunction and its impact on the cardiovascular system, in COVID-19 patients.

## Materials and Methods

In a case control, observational study we analyzed 116 COVID-19 patients (Table 1), admitted from May 11th till June 18th, 2020 at University Clinical Centre of the Republic of Srpska, Bosnia and Herzegovina. During this period, the hospital was able to provide efficient treatment and simultaneously perform signal acquisition for academic purposes. In mid-June, the pandemic escalated in our region and the number of incoming patients exceeded the capacity of the hospital. The number of doctors and nurses became insufficient, so the research activities stopped. For this reason, the number of recorded time series is at the borderline of sufficient sample size.

**TABLE 1:** Population characteristics of COVID-19 patients and controls.

	Mild		Severe		Control	
	F	M	F	M	F	M
Age	46.05 ± 16.78	40.71 ± 16.57	52.18 ± 19.64	51.27 ± 17.60	45.27 ± 18.94	44.11 ± 17.83
Height (cm)	167.35 ± 5.79	181.83 ± 7.95	169.31 ± 6.29	180.37 ± 7.39	168.24 ± 6.31	182.95 ± 7.58
Weight (kg)	65.5 ± 8.46	90.08 ± 15.44	73.40 ± 13.55	87.10 ± 15.25	64.27 ± 11.82	81.27 ± 15.26
BMI (kg/m^2^)	23.36 ± 2.67	27.15 ± 3.57	25.63 ± 4.78	26.66 ± 3.83	22.71 ± 4.08	23.87 ± 3.67

*Results are given as mean ± standard deviation.*

All patients were diagnosed as having COVID-19, according to WHO interim guidance (Berkwits et al., 2020; World Health Organization, 2020a), stating that “the confirmed case of COVID-19 was defined as a positive result on high throughput sequencing or real-time reverse-transcription polymerase chain reaction analysis of throat and nose swab specimens (Berkwits et al., 2020). Throat and nose swab samples were collected and placed into a collection tube containing a preservation solution for the virus (Berkwits et al., 2020). A SARS-CoV-2 infection was confirmed by real-time reverse transcription polymerase chain reaction assay using a SARS-CoV-2 nucleic acid detection kit according to the manufacturer’s protocol (Shanghai bio-germ Medical Technology Co.) (Berkwits et al., 2020). Radiologic assessments included chest CT and all laboratory testing (a complete blood cell count, blood chemical analysis, coagulation testing, assessment of liver and renal function testing, C-reactive protein, creatine kinase, and lactate dehydrogenase) was performed at the admission and repeated according to the clinical care needs of the patient (Berkwits et al., 2020).” Before enrollment, informed consent was obtained from patients. The study was performed in accordance with the principles of the Declaration of Helsinki. This study was approved by the Ethics Committee of University Clinical Centre of the Republic of Srpska, Bosnia and Herzegovina, Number 01-5617.

### Study Protocol

The measurements were performed and the patients checked in the University Clinical Center of the Republic of Srpska following the standard protocol for ANS function and cardiovascular risk assessment.

All patients were tested after clinical stabilization with a negative control PCR test. The study included all patients in a clinically stable condition that allowed testing using cardiovascular reflex tests. Patients with liver and renal disease; with systemic disease (e.g., connective tissue disorders); with a neurological disorder (e.g., cerebrovascular and Parkinson’s disease, Guillain–Barré syndrome, polyneuropathy, multiple sclerosis); with previously existing cardiac diseases (e.g., ischemic or congestive or valvular heart disease, cardiomyopathy, arrhythmia) (Kocabas et al., 2018); patients with a malignancy; were excluded from the analysis. The 75 COVID-19 patients without associated diseases were divided into a severe group (45 patients with confirmed interstitial pneumonia, aged 51.27 ± 19.13, male 24, female 21) and mild group (30 patients, aged 41.56 ± 16.68, male 16, female 14 without pneumonia). Results were compared with 77 healthy, sex and age-matched COVID-19 negative subjects. The patients with associate diseases (CADG) are included in the study and partitioned into a subgroup with diabetes mellitus (CADG-DM, 7 patients), a sub-group with hypertension (CADG-HTA, 18 patients), and a sub-group with syncope (CADG-Syn, 16 patients). Although the sample size of CADG patient groups is not sufficient, they are included in this study for illustrative purposes.

Cardiovascular reflex tests were done between 09:00 and 14:00 a.m., approximately 2 h after light breakfast, under ideal temperature conditions (23°C), without any previous consumption of alcohol, nicotine, or coffee (Ewing and Clarke, 1982; Bellavere et al., 1983; Milovanovic et al., 2011).

### Cardiovascular Reflex Tests (CART)

We performed two parasympathetic tests (heart rate response to Valsalva maneuver, heart rate response to deep breathing) and two tests of sympathetic function (blood pressure response to standing and handgrip test)^2^ :

• **Heart rate response to Valsalva maneuver:** “Valsalva maneuver was performed using a modified sphygmomanometer with blowing and holding pressure of 40 mmHg for 15 s, with ECG recording. The results, expressed as a Valsalva ratio, measured the longest and the shortest RR interval using ruler and electrocardiogram trace^2^.”
• **Deep breathing test:** “Six deep inspirations and expirations were performed over 1 min. The result is expressed as a difference between the highest and the lowest heart rate^2^.”
• **Blood pressure response to standing:** “This test measured the subject’s blood pressure with a sphygmomanometer while the patient was lying quietly and 1 min after the patient was made to stand up. The postural fall in blood pressure was taken as the difference between the systolic pressure lying and the systolic blood pressure standing^2^.” The definition of orthostatic hypotension is as follows: SBP reduction greater than 20 mmHg or DBP reduction greater than 10 mmHg that follows a postural change from supine to standing.
• **The isometric contraction or handgrip test (HG):** “A plastic ball with a medium level of firmness was placed in the right hand of the patient, and the patient was instructed to squeeze and release the ball for 15 s. Then, the patient was instructed to squeeze the ball with the right hand firmly and the test was ended at 1 min (Kocabas et al., 2018).” A rise in BP due to muscular contraction is related to an increase of sympathetic nerve activity at the muscular level. This activity depends both on effort and time. The response of the peripheral alpha sympathetic nerve is presented by the increase of the BP.

According to the systematization and cut-off values proposed by Ewing (Ewing and Clarke, 1982), the results of all five tests are declared as normal, borderline, or abnormal. The patients were categorized as normal if none of the tests was abnormal; with early parasympathetic damage, if results of one of the three tests of parasympathetic function were abnormal; with definite parasympathetic damage, if two or more of the three tests of parasympathetic function were abnormal; and with combined damage, if the test of the sympathetic function was abnormal in addition to parasympathetic damage. For the purpose of the above-mentioned classification, the borderline tests were interpreted as normal. A scoring system, like the one suggested by Bellavere et al. (1983), was also used to assess the extent of autonomic nervous damage.

Heart rate response to standing test (30:15 ratio test) as a measure of parasympathetic and sympathetic activity has not been performed due to technical issues.

### Task Force© Monitor: Beat-to-Beat Analysis of Heart Rate and Blood Pressure Variability and Baroreflex Sensitivity

The ECG and blood pressure waveforms acquisition was performed by Task Force© Monitor (TFM), CNSystems Medizintechnik GmbH, Graz, Austria (CNS) (Kocabas et al., 2018; CNSystems, 2020), which also provides beat-to-beat R–R interval (RRI) and its inverse hear rate (HR) time series, as well as beat-to-beat systolic and diastolic blood pressure (sBP, dBP) by the vascular unloading technique (Gratze et al., 1998; Parati et al., 2003), which was corrected automatically to the oscillometric blood pressure measured on the contralateral arm (Zawadka-Kunikowska et al., 2018).

Task Force© also includes embedded software for power spectral density estimation suitable for non-stationary signals: it implements an adaptive autoregressive (AR) model with a recursive least square algorithm for AR coefficients update (Bianchi, 2011). The software output comprises total power, as well as the powers of very low frequency (VLF), low frequency (LF), and high frequency (HF) bands. The division of the frequency domain is 0–0.04 Hz – VLF band; 0.04–0.15 Hz – LF band; and 0.15–0.40 Hz – HF band). The power spectral density is computed in absolute values (ms^2^ or mmHg^2^ per Hz, depending on whether RRI or SBP are used as the source signal) or normalized units (%) (No authors, 1996). Parameters encompassed into analysis were: LFnu-RRI -- normalized low frequency component of HRV, HFnu-RRI- normalized high frequency component of HRV, VLF-RRI -- very low frequency component of HRV, LF-RRI -- low frequency component of HRV, HF-RRI -- high frequency component of HRV^3^, LF/HF-RRI – low frequency/high frequency ratio of HRV.

It has been shown in some studies (Malliani et al., 1991; Hayano and Yuda, 2019) that the spectral density of cardiovascular signals can be affected by other sources, and that the observed changes are not a consequence of parasympathetic activity only, but also of respiratory movements. The Standards of measurement and physiological interpretation of HRV (No authors, 1996) state that “The efferent vagal activity is a major contributor to the HF component,” that “LF and HF can increase under different conditions” and that “an increase in HF is induced by controlled respiration” with a reference to Malliani et al. (1991).

On the other hand, the CNSystems Medizintechnik GmbH, manufacturer of Task Force© software, does not provide an option to check for the respiratory-induced influence. For this reason, we explored the most recently published scientific papers that implement TFM, available from the CNS. Out of 23 manuscripts, seven used the embedded software to estimate the spectral density and the corresponding power within the characteristic frequency bands.

Controlled respiration concentrates the spectral power in the vicinity of a single frequency, located within the HF band. It was used in two papers. In Kristiansen et al. (2019), in spite of the controlled breathing, it was explicitly stated that “vagal (parasympathetic) activity is the main contributor to HF variability.” In Alvarado-Alvarez et al. (2020) an external spectral analysis was performed using the Hilbert-Huang transform and Empiric mode decomposition. It confirmed that the energy in HF, “due to vagal activity,” is higher in healthy controls than in explored patients, but also that “… the sympathetic modulation of the vasculature is higher than the respiratory influence,” noting the respiration would be taken in the account in the future experiments. Thus also in COVID-19 patients, future studies should take into account the breathing frequency.

The work (Spiesshoefer et al., 2020) was devoted to breathing problems. It was shown that no significant change was observed in sympathovagal balance during the prolonged breathing disturbances in sleep, except for the increased VLF component in one group of patients.

In the case of COVID-19 patients, their overall condition, as well as the following CART test that could have been compromised, prevented the use of the metronome for controlled breathing.

Baroreceptor reflex sensitivity (BRS) is automatically assessed using the sequence technique according to Parati et al. (1995). Beat to beat analysis of blood pressure enables assessment of BRS from spontaneously occurring blood pressure rise and falls which are followed with regulatory heart rate interval changes. Low baroreceptor sensitivity indicates autonomic dysfunction (AD).

### Non-linear Geometric Measures

The Poincaré plot (PPlot) is a scatter plot in which each R–R interval (*y*-axis) is plotted against the previous R–R interval (*x*-axis) (Kamen et al., 1996). The points of the plot are gathered around an identity line. Then an ellipse is fitted, with the center coinciding with the midpoint that corresponds to the average R–R interval. There are two standard deviations (SD) measures, SD1 and SD2, that can be derived.

The ellipse’s width is specified by the standard deviation SD1, calculated as SD of distances from the line of identity. SD1 measures short-term variability, or, more precisely, the variability over a single beat. SD1 is related to the HF spectral components.

The standard deviation SD2 is calculated as the SD of the distances from the line that is perpendicular to the identity line and intersects it at the center point. It measures long-term variability and is related to the LF spectral components. The ratio of standard deviations, SD1/SD2, measures the unpredictability of the R–R intervals.

Besides the quantitative measures, the Poincaré plot is known as a technique for visualization. PPlot of R–R intervals considered as normal are symmetric around the identity line, shaped as fan, comet, or torpedo. Abnormal patterns are characterized by asymmetric configurations, or by narrow configuration (low SD1) of torpedo (and other) shapes.

These derivations are also performed at the “rest” position before the patient was exposed to CART tests.

### Statistical Methods

Results are presented in tables as mean ± standard deviation, or as count (percent), depending on the data type. The results comprising continuous data were tested by the Kolmogorov-Smirnov test for normality. As the results did not follow Gaussian (normal) distribution, the comparisons were made by Kruskal–Wallis non-parametric test followed by Dunn–Bonferroni correction. Bivariate analysis of results comprising categorical data was done by chi-squared test. All data were analyzed using SPSS 15.0 statistical software. The level of significance was set at *p* < 0.05.

## Results

### CART

**FIGURE 1:**
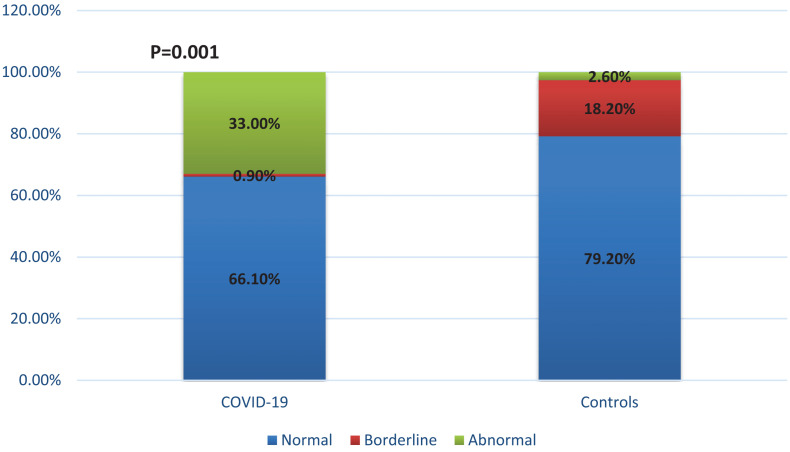
Orthostatic hypotension in COVID-19. Results for COVID-19 patients are given in respect to both severe and mild group.

Blood pressure response to standing revealed OH in 33% of COVID-19 patients (25.0% in severe and in 46.3% mild cases, *p* = 0.001, compared to healthy controls (Figure 1). HG was also more often abnormal in COVID-19 patients, comparing to healthy controls (84.6% in severe and in 94.4% mild cases, *p* = 0.001) leading to the conclusion that impaired sympathetic function of the ANS is significantly more often present in COVID-19 (Table 2, *p* = 0.001).

**TABLE 2:** Abnormal results of cardioreflex tests in COVID-19 based on severity of disease.

	*N* (%)		
	Severe (*n* = 45)	Mild (*n* = 30)	Controls (*n* = 77)
***CART***			
*Sympathetic function tests*			
Blood pressure response to standing (OH)	11 (25.00)***	14 (46.30)***	2 (2.60)
Hand grip test (HG)	38 (84.60)***	28 (94.40)***	59 (76.90)
***Parasympathetic function tests***			
Heart rate response to Valsalva maneuver	8 (18.20)***	7 (28.90)**	19 (24.40)
Heart rate response to deep breathing	12 (25.80)***	12 (41.70)***	9 (11.50)
***ANS impairment***			
Parasympathetic dysfunction	5 (12.10)**	8 (26.60)*	11 (14.10)
Combined dysfunction	24 (53.50)***	22 (73.20)***	9 (11.80)
Autonomic neuropathy	23 (51.50)*	23 (76.70)*	43 (55.80)

*CART, cardioreflex test; ANS, autonomic nervous system. Results are presented as counts (percent).*

Bivariate analysis of severe and mild group with respect to controls is done by chi-squared test.

*p ≤ 0.05, **p ≤ 0.01, ***p ≤ 0.001.

Tests for parasympathetic activity evaluation were abnormal in some COVID-19 patients, as presented in Table 2. Valsalva maneuver was found abnormal in 18.2% severe and 28.9% mild COVID-19 cases, compared to 24.4% abnormal findings in healthy controls (*p* = 0.003). Heart rate response to deep breathing was abnormal in 25.8% severe and 41.7% COVID-19 patients and that was highly significantly more often compared to controls (*p* = 0.001).

Hence, in COVID-19, significant impairment of parasympathetic activity has been detected, leading to significant combined sympathetic and parasympathetic AD in COVID-19, as presented in Table 2. AD (sympathetic, parasympathetic, or both) in our study has been revealed in 51.5% of severe, 76.7% of mild COVID-19 patients, and the difference compared to healthy controls was significant (*p* = 0.018).

### Beat to Beat Task Force Monitor Analysis

#### HRV

As presented in Table 3, the heart rate (HR) was significantly higher in COVID-19 patients during the first, “resting,” phase of the measurements. There was no difference regarding levels of systolic and diastolic blood pressure values between COVID-19 patients and controls. HRV measurements on Task Force Monitor revealed a moderate but statistically insignificant increase and decrease of VLF-RRI components in severe and mild COVID-19 patients, respectively. The Low-frequency component of HRV (LF-RRI), as a marker of sympathetic and parasympathetic activity, was significantly lower in COVID-19 patients, most prominently in the severe presentation of the disease. The same goes for the high-frequency component of HRV (HF-RRI) of mild COVID-19 patients. LF/HF-RRI ratio was significantly higher in severe COVID-19 patients, implying higher sympathetic activity of ANS.

**TABLE 3:** Beat-to-beat analysis of heart rate variability (Task force monitor) in COVID-19.

Parameter	Severe (*n* = 45)	Mild (*n* = 30)	Controls (*n* = 77)
***Beat statistics***			
HR (bpm)	82.57 ± 16.71*	81.86 ± 13.60*	72.30 ± 9.96
SBP (mmHg)	113.84 ± 24.26	112.08 ± 13.18	116.01 ± 13.28
DBP (mmHg)	82.01 ± 22.26	72.71 ± 12.29	77.17 ± 10.27
***HRV statistics***			
LFnu-RRI (%)	65.81 ± 21.43	58.24 ± 22.81	60.05 ± 15.88
HFnu-RRI (%)	34.68 ± 24.85	39.70 ± 14.79	39.68 ± 15.27
VLF-RRI (msec^2^)	639.51 ± 3232.01	238.69 ± 376.95	500.73 ± 842.22
LF-RRI (msec^2^)	449.40 ± 556.43*	414 ± 460.27*	833.05 ± 964.79
HF-RRI (msec^2^)	483.89 ± 1214.17	439.38 ± 314.78*	607.19 ± 836.32
LF/HF-RRI	4.89 ± 6.54*	3.21 ± 2.72	2.81 ± 2.57
***Non-linear measurements***			
SD1	48.19 ± 48.51	31.15 ± 21.55*	41.09 ± 25.76
SD2	84.47 ± 48.79	81.86 ± 31.47	82.71 ± 34.93
SD1/SD2	0.52 ± 0.33	0.37 ± 0.21*	0.48 ± 0.22

*HR, heart rate; SBP, systolic blood pressure; DBP, diastolic blood pressure; HRV, heart rate variability; LFnu-RRI, normalized low frequency component of HRV; HFnu-RRI, normalized high frequency component of HRV; VLF-RRI, very low frequency component of HRV; LF-RRI, low frequency component of HRV; HF-RRI, high frequency component of HRV; LF/HF-RRI, low frequency/high freqeuency ratio of HRV.*

*Results are presented as mean ± standard deviation (SD).*

Statistical significance is assessed by Kruskal–Wallis non-parametric test followed by Dunn’s test with Bonferroni correction, ‘*’ denotes *p* < 0.05 with respect to the control group.

#### Systolic BPV

Among markers of ANS systolic blood pressure modulation, HF-nu sBP was significantly higher in mild COVID-19 patients, compared to healthy subjects. This marker is associated with parasympathetic activity, but also to the mechanical effects of respiration. The same parameters in severe patients have also increased and decreased but without statistical significance. There was no significant difference regarding other parameters of systolic BPV analyzed.

#### Diastolic BPV

Assessment of diastolic BPV, as presented in Table 4, revealed lower levels of sympathetic activity marker (LF-nu dBP) and higher levels of parasympathetic activity marker (HF-nu dBP) in COVID-19 patients. LF/HFdBP ratio was lower, implying a higher parasympathetic tone in both groups of COVID-19 patients. VLFdBP was higher, especially in severe COVID-19 patients compared to healthy subjects, as presented in Table 4, but with a large standard deviation and without statistical significance.

**TABLE 4:** Beat-to-beat analysis of blood pressure variability and baroreceptor reflex sensitivity (Task force monitor) in COVID-19.

Parameter	Severe (*n* = 45)	Mild (*n* = 30)	Controls (*n* = 77)
***BPV (systolic) statistics***			
LFnu-sBP (%)	42.13 ± 17.35	42.44 ± 20.56	50.83 ± 13.13
HFnu-sBP (%)	16.32 ± 10.48	20.73 ± 11.35*	13.94 ± 7.27
VLF-sBP	5.28 ± 11.09	12.11 ± 62.96	3.21 ± 3.01
LF-sBP	3.29 ± 3.63	5.62 ± 6.15	4.853 ± 7.08
HF-sBP	1.24 ± 1.29	1.68 ± 2.39	0.973 ± 1.41
LF/HF-sBP	3.37 ± 2.67	2.81 ± 3.21*	4.12 ± 4.32
***BPV (diastolic) statistics***			
LFnu-dBP (%)	44.67 ± 16.35*	39.28 ± 17.34*	50.12 ± 10.51
HFnu-dBP (%)	15.98 ± 10.64*	17.29 ± 10.03*	13.46 ± 8.49
VLF-dBP	29.34 ± 99.93	11.56 ± 60.59	4.88 ± 4.85
LF-dBP	11.41 ± 24.89	12.01 ± 17.88	6.73 ± 12.96
HF-dBP	4.25 ± 11.33	3.55 ± 4.08	1.79 ± 2.06
LF/HF-dBP	3.92 ± 2.06*	3.71 ± 2.02*	6.21 ± 3.07
***BRS***			
Slope mean	13.83 ± 12.54*	11.16 ± 7.77*	17.24 ± 9.87
BEI	52.03 ± 22.94*	44.63 ± 25.69*	119.57 ± 43.43

*BPV, blood pressure variability; LFnu-dBP, normalized low frequency component of BPV; HFnu-dBP, normalized high frequency component of BPV; VLF-dBP, very low frequency component of BPV; LF-dBP, low frequency component of BPV; LF/HF-dBP, low frequency/high freqeuency ratio of BPV; BRS, baroreceptor reflex sensitivity; BEI, baroreflex efficacy index.*

*Results are presented as mean ± standard deviation (SD).*

Statistical significance is assessed by Kruskal–Wallis non-parametric test followed by Dunn’s test with Bonferroni correction, ‘*’ denotes *p* < 0.05 with respect to the control group.

#### Baroreflex Sensitivity (BRS)

**FIGURE 2:**
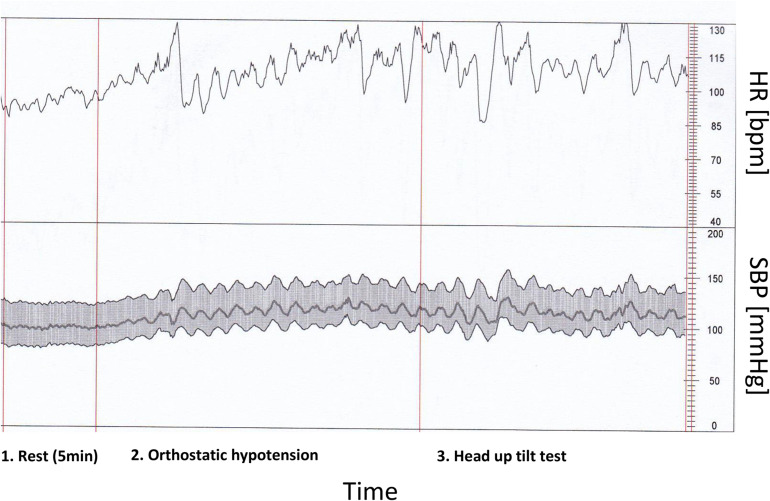
Heart rate and characteristic blood pressure variability in rest and during orthostatic hypotension and head up tilt test by patient with COVID-19 infection and low baroreflex sensitivity (real time beat to beat blood pressure analysis).

Mean slope (BRS) and baroreflex effectiveness index (BEI) revealed significantly lower values in COVID-19, as presented in Table 4. It can be also concluded from a real-time beat to beat blood pressure analysis presented in Figure 2.

#### Non-linear Measurements

SD1, SD2, as well as SD1/SD2 ratio of Poincaré plot in severe COVID-19 patients has not changed with respect to controls (Table 3). However, SD1 and, consequently, SD1/SD2 ratio was significantly lower in mild COVID-19 patients with respect to controls.

**FIGURE 3:**
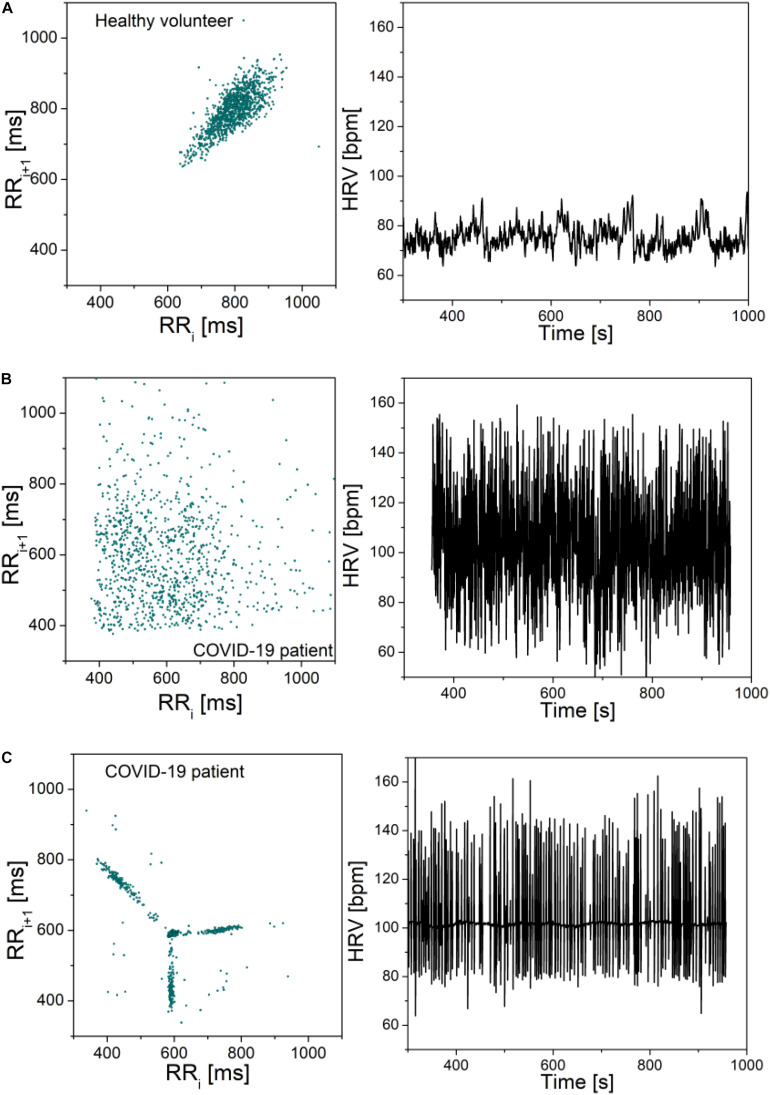
Poincare plots and corresponding heart rate signals: **(A)** Healthy volunteer; **(B)** COVID-19 patient; **(C)** COVID-19 patient. Note that the scale is the same in all graphs. The plots are included to illustrate the adverse effects of COVID-19 infection, but the signals were not part of the presented statistics. Patient (b) is male, 71 years old, height 168, weight 66. The patient reported no hereditary diseases, gait instability, and last 2 months the patient was experiencing hard breathing. During hospitalization it was discovered that the patient has heart valve disease, before that the patient was healthy. Patient (c) is female, 87 years old, height 163, weight 81. She reported problems with spine, occasional headaches, dizziness when changing her head position, no hereditary diseases and no other health problems.

To illustrate the severity of COVID-19 infection, Figure 3 presents the Poincare plots of the excluded patients, together with their heart rate beat-to-beat time series. Healthy control is presented as well, for the sake of comparison.

### Assessment of ANS Function in COVID-19 With Associated Diseases

#### CART

As presented in Table 5, CADG patients had significantly impaired results of almost all CART tests implied. OH was revealed in 57.1% of CADG-DM and in 52.9% of CADG-HTA patients with a highly statistically significant difference, compared to the CG group and healthy controls (*p* = 0.001 for both). AD was established in 78.0% of overall CADG patients, 83.3% of diabetics with COVID-19, in 82.4% of patients with hypertension and COVID-19, and 66.7% of CADG-Syn patients, and the difference with respect to the control group was significant for the CADG group (*p* = 0.018, *p* = 0.315, *p* = 0.069, *p* = 0.552, respectively).

**TABLE 5:** Abnormal results of cardioreflex tests in different subgroups of COVID-19 patients with associated co-morbidities.

	*N* (%)							
	CADG (*n* = 41)	*p*-value	CADG-DM (*n* = 7)	*p*-value	CADG-HTA (*n* = 18)	*p*-value	CADG-Syn (*n* = 16)	*p*-value
***CART***								
***Sympathetic function tests***								
Blood pressure response to standing (OH)	19 (46.30)	0.001	4 (57.10)	0.001	10 (52.90)	0.001	5 (31.20)	0.001
Hand grip test (HG)	39 (94.40)	0.001	7 (100.00)	0.001	18 (100.00)	0.001	15 (93.70)	0.001
***Parasympathetic function tests***								
Heart rate response to Valsalva maneuver	12 (28.90)	0.001	3 (40.00)	0.001	5 (27.70)	0.001	6 (38.50)	0.001
Heart rate response to deep breathing	17 (41.70)	0.001	3 (40.00)	0.001	8 (46.70)	0.001	4 (25.00)	0.001
***ANS impairment***								
Parasympathetic dysfunction	10 (24.00)	0.057	3 (40.00)	0.097	4 (22.20)	0.131	1 (6.20)	0.282
Combined dysfunction	30 (73.20)	0.001	7 (100.00)	0.001	15 (82.40)	0.001	10 (60.00)	0.001
Autonomic neuropathy	32 (78.00)	0.018	6 (83.30)	0.315	15 (82.40)	0.069	11 (66.70)	0.552

*CART, cardioreflex test; CADG, COVID-19 group with associated diseases; CADG-DM, COVID-19 group with diabetes mellitus; CADG-HTA, COVID-19 group with hypertension; CADG-Syn, COVID-19 group with syncope.*

*Results are presented as counts (percent).*

*Bivariate analysis of severe and mild group with respect to controls is done by chi-squared test.*

Separate analysis has been performed for each co-morbidity in COVID-19 patients and results were presented in Tables 6–9.

**TABLE 6:** HRV and BPV in COVID-19 group with associated diseases (CADG).

Parameter	CADG (*n* = 41)	CG (*n* = 75)	Controls (*n* = 77)
***Beat statistics***			
HR (bpm)	80.83 ± 14.13*	83.16 ± 16.30*	72.304 ± 9.95
SBP (mmHg)	112.39 ± 17.99	113.67 ± 22.47	116.010 ± 13.28
DBP (mmHg)	72.91 ± 13.26	82.92 ± 81.95	77.171 ± 10.26
***HRV statistics***			
LFnu-RRI (%)	58.24 ± 22.80	58.09 ± 21.42	60.050 ± 15.88
HFnu-RRI (%)	39.46 ± 22.41	34.20 ± 21.42	39.675 ± 15.27
VLF-RRI (msec^2^)	328.26 ± 643.28	639.81 ± 3263.88	500.73 ± 842.21
LF-RRI (msec^2^)	414.21 ± 559.96*	449.95 ± 497.03*	833.05 ± 964.79
HF-RRI (msec^2^)	439.79 ± 775.86	483.58 ± 1092.56	607.19 ± 836.32
LF/HF-RRI	3.267 ± 4.24	4.89 ± 6.74*	2.85 ± 3.71
***Non-linear measurements***			
SD1	44.40 ± 39.36	39.80 ± 41.98	41.09 ± 25.76
SD2	81.56 ± 46.80	84.58 ± 40.38	82.71 ± 34.93
SD1/SD2	0.50 ± 0.24	0.43 ± 0.32	0.48 ± 0.21
***BPV (systolic) statistics***			
LFnu-sBP (%)	38.31 ± 16.75	41.50 ± 16.77	45.38 ± 10.51
HFnu-sBP (%)	20.36 ± 11.75*	16.37 ± 9.57	13.45 ± 8.48
VLF-sBP	11.60 ± 19.76	29.55 ± 107.44	4.87 ± 4.84
LF-sBP	9.67 ± 14.21	12.81 ± 25.93	6.72 ± 12.96
HF-sBP	5.22 ± 13.65	3.21 ± 4.60	1.79 ± 2.06
LF/HF-sBP	2.80 ± 2.14*	3.37 ± 1.96	4.11 ± 3.07
***BPV (diastolic) statistics***			
LFnu-dBP (%)	39.12 ± 20.11*	44.13 ± 17.53	50.83 ± 13.13
HFnu-dBP (%)	17.29 ± 13.26*	15.98 ± 9.49	11.947 ± 7.26
VLF-dBP	5.17 ± 6.66	12.30 ± 51.43	3.214 ± 3.01
LF-dBP	3.37 ± 3.44	4.73 ± 5.57	4.853 ± 7.07
HF-dBP	1.31 ± 1.50	1.47 ± 1.99	0.973 ± 1.39
LF/HF-dBP	3.72 ± 3.18*	3.90 ± 2.81*	6.21 ± 4.32
***BRS***			
Slope mean	11.16 ± 9.28*	13.83 ± 11.75	17.24 ± 9.86
BEI	44.24 ± 24.25*#	52.90 ± 23.70*	119.57 ± 43.42

*CADG, COVID-19 group with associated diseases, CG – COVID-19 group without comorbidities; BPV, blood pressure variability; LFnu-dBP, normalized low frequency component of BPV; HFnu-dBP, normalized high frequency component of BPV; VLF-dBP, very low frequency component of BPV; LF-dBP, low frequency component of BPV; LF/HF-dBP, low frequency/high freqeuency ratio of BPV; BRS, baroreceptor reflex sensitivity; BEI, baroreflex efficacy index.*

*Results are presented as mean ± standard deviation (SD).*

Statistical significance is assessed by Kruskal–Wallis non-parametric test followed by Dunn’s test with Bonferroni correction, ‘*’ denotes *p* < 0.05 with respect to the controls, ‘#’ with respect to CG.

**TABLE 7:** HRV and BPV in COVID-19 group with diabetes mellitus (CADG-DM).

Parameter	CADG-DM (*n* = 7)	CG (*n* = 75)	Controls (*n* = 77)
***Beat statistics***			
HR (bpm)	92.90 ± 13.65*	83.16 ± 16.30*	72.304 ± 9.95
SBP (mmHg)	130.74 ± 21.72*	113.67 ± 22.47	116.010 ± 13.28
DBP (mmHg)	74.68 ± 7.11	82.92 ± 81.95	77.171 ± 10.26
***HRV statistics***			
LFnu-RRI (%)	55.92 ± 27.95	5.809 ± 21.4252	60.050 ± 15.88
HFnu-RRI (%)	44.07 ± 27.95	34.200 ± 21.42	39.675 ± 15.27
VLF-RRI (msec^2^)	101.14 ± 117.65	639.81 ± 3263.88	500.73 ± 842.21
LF-RRI (msec^2^)	230.71 ± 253.18*#	449.95 ± 497.03*	833.05 ± 964.79
HF-RRI (msec^2^)	389.57 ± 644.85	483.58 ± 1092.56	607.19 ± 836.32
LF/HF-RRI	4.41 ± 5.87	4.89 ± 6.74*	2.85 ± 3.71
***Non-linear measurements***			
SD1	51.75 ± 44.30	39.80 ± 41.98	41.09 ± 25.76
SD2	71.63 ± 39.62	84.58 ± 40.38	82.71 ± 34.93
SD1/SD2	0.63 ± 0.27	0.43 ± 0.32	0.48 ± 0.21
***BPV (systolic) statistics***			
LFnu-sBP (%)	38.50 ± 13.38	41.50 ± 16.77	45.38 ± 10.51
HFnu-sBP (%)	13.38 ± 9.56	16.37 ± 9.57	13.45 ± 8.48
VLF-sBP	3.61 ± 3.81	29.55 ± 107.44	4.87 ± 4.84
LF-sBP	3.56 ± 4.74	12.81 ± 25.93	6.72 ± 12.96
HF-sBP	1.68 ± 1.79	3.21 ± 4.60	1.79 ± 2.06
LF/HF-sBP	2.08 ± 0.93	3.37 ± 1.96	4.11 ± 3.07
***BPV (diastolic) statistics***			
LFnu-dBP (%)	34.11 ± 15.72*	44.13 ± 17.53	50.83 ± 13.13
HFnu-dBP (%)	17.40 ± 13.69	15.98 ± 9.49	11.947 ± 7.26
VLF-dBP	3.43 ± 4.46	12.30 ± 51.43	3.214 ± 3.01
LF-dBP	1.56 ± 2.08	4.73 ± 5.57	4.853 ± 7.07
HF-dBP	0.80 ± 1.23	1.47 ± 1.99	0.973 ± 1.39
LF/HF-dBP	3.08 ± 2.51*	3.90 ± 2.81*	6.21 ± 4.32
***BRS***			
Slope mean	10.14 ± 10.71	13.83 ± 11.75	17.24 ± 9.86
BEI	35.85 ± 21.16*	52.90 ± 23.70*	119.57 ± 43.42

*CADG-DM, COVID-19 group associated with diabetes mellitus; CG, COVID-19 group without comorbidities; BPV, blood pressure variability; LFnu-dBP, normalized low frequency component of BPV; HFnu-dBP, normalized high frequency component of BPV; VLF-dBP, very low frequency component of BPV; LF-dBP, low frequency component of BPV; LF/HF-dBP, low frequency/high freqeuency ratio of BPV; BRS, baroreceptor reflex sensitivity; BEI, baroreflex efficacy index.*

*Results are presented as mean ± standard deviation (SD).*

Statistical significance is assessed by Kruskal–Wallis non-parametric test followed by Dunn’s test with Bonferroni correction, ‘*’ denotes *p* < 0.05 with respect to the controls, ‘#’ with respect to CG.

**TABLE 8:** HRV and BPV in COVID-19 group with hypertension (CADG-HTA).

Parameter	CADG-HTA (*n* = 18)	CG (*n* = 75)	Controls (*n* = 77)
***Beat statistics***			
HR (bpm)	80.05 ± 14.39*	83.16 ± 16.30*	72.304 ± 9.95
SBP (mmHg)	113.90 ± 24.24	113.67 ± 22.47	116.010 ± 13.28
DBP (mmHg)	70.37 ± 15.87	82.92 ± 81.95	77.171 ± 10.26
***HRV statistics***			
LFnu-RRI (%)	58.56 ± 23.10	5.809 ± 21.4252	60.050 ± 15.88
HFnu-RRI (%)	41.43 ± 23.10	34.200 ± 21.42	39.675 ± 15.27
VLF-RRI (msec^2^)	401.11 ± 911.65	639.81 ± 3263.88	500.73 ± 842.21
LF-RRI (msec^2^)	316.17 ± 610.83*	449.95 ± 497.03*	833.05 ± 964.79
HF-RRI (msec^2^)	477.00 ± 917.58	483.58 ± 1092.56	607.19 ± 836.32
**LF/HF-RRI**	2.77 ± 3.21	4.89 ± 6.74*	2.85 ± 3.71
***Non-linear measurements***			
SD1	41.19 ± 37.13	39.80 ± 41.98	41.09 ± 25.76
SD2	69.32 ± 35.32	84.58 ± 40.38	82.71 ± 34.93
SD1/SD2	0.52 ± 0.25	0.43 ± 0.32	0.48 ± 0.21
***BPV (systolic) statistics***			
LFnu-sBP (%)	35.98 ± 20.93	41.50 ± 16.77	45.38 ± 10.51
HFnu-sBP (%)	19.81 ± 12.46*	16.37 ± 9.57	13.45 ± 8.48
VLF-sBP	16.24 ± 27.27	29.55 ± 107.44	4.87 ± 4.84
LF-sBP	10.12 ± 15.50	12.81 ± 25.93	6.72 ± 12.96
HF-sBP	3.16 ± 2.43	3.21 ± 4.60	1.79 ± 2.06
LF/HF-sBP	2.95 ± 2.13	3.37 ± 1.96	4.11 ± 3.07
***BPV (diastolic) statistics***			
LFnu-dBP (%)	41.06 ± 23.02	44.13 ± 17.53	50.83 ± 13.13
HFnu-dBP (%)	12.55 ± 9.27	15.98 ± 9.49	11.947 ± 7.26
VLF-dBP	4.49 ± 6.12	12.30 ± 51.43	3.214 ± 3.01
LF-dBP	3.67 ± 3.81	4.73 ± 5.57	4.853 ± 7.07
HF-dBP	1.10 ± 1.03	1.47 ± 1.99	0.973 ± 1.39
LF/HF-dBP	4.57 ± 3.47*	3.90 ± 2.81*	6.21 ± 4.32
***BRS***			
Slope mean	8.85 ± 7.57*#	13.83 ± 11.75*	17.24 ± 9.86
BEI	40.55 ± 20.76*	52.90 ± 23.70*	119.57 ± 43.42

*CADG-HTA, COVID-19 group associated with hypertension; CG, COVID-19 group without comorbidities; BPV, blood pressure variability; LFnu-dBP, normalized low frequency component of BPV; HFnu-dBP, normalized high frequency component of BPV; VLF-dBP, very low frequency component of BPV; LF-dBP, low frequency component of BPV; LF/HF-dBP, low frequency/high freqeuency ratio of BPV; BRS, baroreceptor reflex sensitivity; BEI, baroreflex efficacy index.*

*Results are presented as mean ± standard deviation (SD).*

Statistical significance is assessed by Kruskal–Wallis non-parametric test followed by Dunn’s test with Bonferroni correction, ‘*’ denotes *p* < 0.05 with respect to the controls, ‘#’ with respect to CG.

**TABLE 9:** HRV and BPV in COVID-19 group with syncope (CADG-Syn).

Parameter	CADG-Syn (*n* = 16)	CG (*n* = 75)	Controls (*n* = 77)
***Beat statistics***			
HR (bpm)	83.93 ± 9.96*	83.16 ± 16.30*	72.304 ± 9.95
SBP (mmHg)	116.01 ± 13.28	113.67 ± 22.47	116.010 ± 13.28
DBP (mmHg)	82.01 ± 22.26	82.92 ± 81.95	77.171 ± 10.26
***HRV statistics***			
LFnu-RRI (%)	60.27 ± 21.29	5.809 ± 21.4252	60.050 ± 15.88
HFnu-RRI (%)	36.37 ± 20.98	34.200 ± 21.42	39.675 ± 15.27
VLF-RRI (msec^2^)	393.94 ± 804.37	639.81 ± 3263.88	500.73 ± 842.21
LF-RRI (msec^2^)	385.75 ± 427.64*	449.95 ± 497.03*	833.05 ± 964.79
HF-RRI (msec^2^)	243.56 ± 315.54	483.58 ± 1092.56	607.19 ± 836.32
**LF/HF-RRI**	3.675 ± 5.40	4.89 ± 6.74*	2.85 ± 3.71
***Non-linear measurements***			
SD1	43.89 ± 50.12	39.80 ± 41.98	41.09 ± 25.76
SD2	86.98 ± 59.37	84.58 ± 40.38	82.71 ± 34.93
SD1/SD2	0.45 ± 0.28	0.43 ± 0.32	0.48 ± 0.21
***BPV (systolic) statistics***			
LFnu-sBP (%)	40.39 ± 18.19	41.50 ± 16.77	45.38 ± 10.51
HFnu-sBP (%)	20.70 ± 11.94*	16.37 ± 9.57	13.45 ± 8.48
VLF-sBP	11.37 ± 14.50	29.55 ± 107.44	4.87 ± 4.84
LF-sBP	14.86 ± 21.14	12.81 ± 25.93	6.72 ± 12.96
HF-sBP	9.00 ± 21.67*#	3.21 ± 4.60	1.79 ± 2.06
LF/HF-sBP	2.75 ± 2.13	3.37 ± 1.96	4.11 ± 3.07
***BPV (diastolic) statistics***			
LFnu-dBP (%)	41.65 ± 20.87*	44.13 ± 17.53	50.83 ± 13.13
HFnu-dBP (%)	17.18 ± 11.21*	15.98 ± 9.49	11.947 ± 7.26
VLF-dBP	5.48 ± 7.67	12.30 ± 51.43	3.214 ± 3.01
LF-dBP	4.09 ± 4.09	4.73 ± 5.57	4.853 ± 7.07
HF-dBP	1.43 ± 1.45	1.47 ± 1.99	0.973 ± 1.39
**LF/HF-dBP**	3.67 ± 2.99*	3.90 ± 2.81*	6.21 ± 4.32
***BRS***			
Slope mean	10.53 ± 7.69	13.83 ± 11.75	17.24 ± 9.86
BEI	53.24 ± 23.92*	52.90 ± 23.70*	119.57 ± 43.42

*CADG-Syn, COVID-19 group associated with syncope; CG, COVID-19 group without comorbidities; BPV, blood pressure variability; LFnu-dBP, normalized low frequency component of BPV; HFnu-dBP, normalized high frequency component of BPV; VLF-dBP, very low frequency component of BPV; LF-dBP, low frequency component of BPV; LF/HF-dBP, low frequency/high freqeuency ratio of BPV; BRS, baroreceptor reflex sensitivity; BEI, baroreflex efficacy index.*

*Results are presented as mean ± standard deviation (SD).*

Statistical significance is assessed by Kruskal–Wallis non-parametric test followed by Dunn’s test with Bonferroni correction, ‘*’ denotes *p* < 0.05 with respect to with respect to the controls, ‘#’ with respect to CG.

#### HRV

As presented in Tables 6–9, in a separate HRV analysis of the CADG groups, HR had significantly higher values compared to healthy controls in all co-morbidity groups. Values were less prominent than in CG. Sympathetic activity in CADG, and to a lesser extent in CG, was decreased. It was confirmed through a significantly lower level of LF HRV in the CADG group, with respect to the control group. VLF and HF HRV parameters were also decreased but without statistical significance.

#### BPV

Spectral analysis of diastolic and systolic blood pressure variability in CADG revealed a notable predominance of parasympathetic activity. Values of LF/HFdBP significantly decreased between CADG groups and controls (Tables 6–9). A significant difference with respect to controls was pronounced also for LFnu DBP (CADG, CADG-DM, CADG-Syn), HFnu DBP (CADG, CADH-Syn), HFnu SBP (CADG, CADG-HTA, CADH-Syn), HF SBP (CADG-Syn. BRS parameters were also significantly lower in the CADG group, slope means in CADG and CADG- HTA, while BEI also significantly decreased in all four groups, implying a higher sudden cardiac death risk in this population of patients.

There was no significant difference in any of the Poincaré plot parameters in CADG with respect to controls.

## Discussion

We found evidence of cardiac AD in patients with COVID-19. This study adds to the accumulating evidence COVID-19 affects autonomic nerves and this may explain some of its clinical features namely orthostatic intolerance syndrome. Exclusion criteria were strict, allowing more certainty of the results presented. Although the numbers were small, we have demonstrated significant abnormalities in autonomic function between controls and patients, using basic CART (Rogstad et al., 1999).

For more than a half of patients analyzed, we established the loss of sympatho-vagal ANS balance in COVID-19 patients, in the means of sympathetic and parasympathetic dysfunction. OH as a cardinal sign of sympathetic dysfunction existed in about a third of patients with COVID-19 infection, but also in half of the patients with diabetes and slightly more than half in patients with hypertension. Disorder of baroreflex activity and reduced baroreflex sensitivity with high statistical significance is a key finding in this group of patients with marked pressure variability. However, it has been shown that, during HG tests, many patients actually compromise the results by performing a Valsalva maneuver (Hilz and Dütsch, 2006; Zygmunt and Stanczyk, 2010). This test is even proposed (Körei et al., 2017) to be excluded from cardiovascular autonomic testing, as its results do not show association with those of the other Ewing and Clarke tests. It was also reported (Mao et al., 2020) that HG highly depends both on hypertensive status, and baseline dBP of the patient. But, this is still a standardized part of Ewing and Clarke’s battery of five tests (Ewing and Clarke, 1982; Freeman and Chapleau, 2013) and we opted not to exclude it. Since all the measurements were performed in equal conditions, the increase of abnormal HG in COVID-19 patients was still pronounced.

The CART findings were confirmed in HRV and BPV analysis on Task Force Monitor. The lower sympathetic activity revealed onto various markers analyzed in the modulation of systolic blood pressure, followed by a higher parasympathetic tone, could be explained by compensatory mechanism or a result of sympathetic dysfunction in COVID-19.

Non-linear HRV analysis using the Poincare plots, considered as the simplest SCD risk predictors, revealed a statistically significant parametric difference in COVID-19.

Dysfunction of both parts of the ANS, including vagal and sympathetic activity, the occurrence of OH in a high percentage, decreased baroreflex sensitivity, and changes in the structure of the Poincare shape are cardinal signs of increased risk in these patients for multisystemic disorders. Chronic fatigue syndrome, an entity that is already taking on epidemic proportions after infection with viruses and especially with COVID-19 is one of the complications. Evaluation in this direction is warranted in further studies, especially in correlation with the degree of AD.

All markers of diastolic BPV implied lower sympathetic and compensatory higher parasympathetic activity in the modulation of diastolic blood pressure. BRS analysis also confirmed significant impairment of sympathetic tone.

The pronounced abnormalities were confirmed comparing COVID-19 patients to healthy subjects, with an increase of AD; from asymptomatic to COVID-19 patients with pneumonia (defined as severe cases). This shows that dysfunction can occur at any stage of the disease, including patients with mild symptoms.

Heart rate response to deep breathing was abnormal in 25.8% severe and 41.7% COVID-19 patients and that was highly significantly more often compared to controls. Heart response to standing test could not be easily performed by COVID-19 patients because of technical reasons and these results were not taken into account. Heart rate was significantly higher in COVID-19 patients. Its high standard deviation, despite the statistical significance, indicates that a few patients exhibited an opposite effect. Such variability was observed in a range of features and is a characteristic of COVID-19 patients. There was no difference regarding levels of systolic and diastolic blood pressure values between COVID-19 patients and controls. HRV measurements on Task Force Monitor revealed a moderate but statistically insignificant increase and decrease of VLF-RRI components in severe and mild COVID-19 patients, respectively. The Low-frequency component of HRV (LF-RRI), as a marker of sympathetic and parasympathetic activity, was significantly lower in COVID-19 patients, most prominently in the severe presentation of the disease. The same goes for the high-frequency component of HRV (HF-RRI) of mild COVID-19 patients, while in severe patients, although the mean value is obviously decreased, a large standard deviation testifies to the large variability of the HF-RRI component in this group of patients. LF/HF-RRI ratio was significantly higher in severe COVID-19 patients, implying higher sympathetic activity of ANS.

Several decades after confirmation of ANS affection in viral infection such as HIV, with a confirmed association between inflammation and cardiovascular autonomic neuropathy (CAN), the number of SARS-CoV-2 neurologic manifestations is speedily growing. In a review of 214 patients hospitalized in three dedicated COVID-19 hospitals in Wuhan China, 36% of patients had nervous system symptoms (Koralnik and Tyler, 2020; Mao et al., 2020). Ghosh et al. (2020) described a case of acute onset dysautonomia as a sign of acute motor axonal neuropathy during SARS-CoV-2 infection. Many negative factors, such as the globalization of the infection and multidimensional pathogenic mechanisms have influenced COVID-19 to become a universal threat to the complete nervous system. Despite the current partial understanding of the neuropathogenesis of SARS-CoV-2, our knowledge is growing more and more every day. Direct neuronal invasion by hematogenous or retrograde neuronal route of SARS-CoV-2, similar to SARS and MERS viruses, could be a reasonable pathologic mechanism. Along with inflammatory response and hypercoagulation, damage to ANS could be explained.

Cardiovascular autonomic neuropathy might be an explanation for common cardiovascular manifestations found in COVID-19 patients such as cardiac arrhythmias and cardiac arrest. One of the presenting symptoms in 7.3% of patients admitted for COVID-19 is a non-specific heart palpitation, according to a cohort of 137 patients (Arentz et al., 2020; Kwenandar et al., 2020). These symptoms are more common in ICU patients compared to the non-ICU patients (44.4% vs. 6.9%) although specific types of arrhythmia are not described (Kwenandar et al., 2020; Wang et al., 2020). Although the prevalence of arrhythmia might be attributable to metabolic abnormalities, hypoxia, neurohormonal or inflammatory stress in the settings of viral infection whether the patient has preexisting cardiovascular disease or not, we think that CAN could also be a reasonable cause (Wang et al., 2020). Recently, Del Rio et al. (2020) also hypothesized that promoted resting sympathetic activity along with hypoxemia and decreased parasympathetic activity, could amplify existing proarrhythmic substrate in COVID-19 patients.

Cardiovascular autonomic neuropathy is broadly described as a common and deadly complication of diabetes mellitus, but with pathophysiology remaining with lack of clarity (Fisher and Tahrani, 2017; Liu et al., 2020). Bhati et al. (2019), in their recent study, confirmed the relationship between biomarkers of inflammation to the measures of cardiac vagal tone and HRV, linking the subclinical inflammation to CAN presentation. von Känel et al. (2008), with his study team, described a significant association between systemic low-grade inflammatory activity and decrease in HRV in healthy subjects, confirming a positive relationship between plasma levels of interleukin (IL)-6 and soluble tissue factor (sTF), when HRV was low^4^.

Inflammation and vagally mediated HRV have been implicated in a multitude of disorders including metabolic syndrome and cardiovascular disease (Lau et al., 2005; Thayer et al., 2010; Jarczok et al., 2014). It is generally accepted that the ANS plays an important role in immune function (Tracey, 2010; Luft, 2012; Jarczok et al., 2014). The inflammatory reflex is a physiological mechanism through which the vagus nerve regulates immune function. Accordingly, efferent vagal activity inhibits the release of pro-inflammatory cytokines via the release of acetylcholine and this physiological mechanism has been termed the cholinergic anti-inflammatory pathway^5^ (Pavlov and Tracey, 2012; Matteoli and Boeckxstaens, 2013). In a prospective study of Jarczok et al. (2014), cardiac vagal modulation at baseline predicted level of CRP 4 years later (Craddock et al., 1987).

It was Craddock with his associates who described the first abnormalities of the ANS in HIV infection^6^ (Craddock et al., 1987). Subsequently, Rogstad et al. (1999), in their prospective, case-control study, reported abnormalities in autonomic function in HIV-positive patients, symptomatic as well as asymptomatic. The pathogenesis of CAN in HIV was incompletely understood. There is sympathetic dysfunction of lymph nodes in the rhesus macaque following acute HIV infection (Sloan et al., 2008; Robinson-Papp et al., 2013) but lymph nodes contain a high concentration of virally infected cells and it is unknown if autonomic innervation of other organs is similarly affected. Autonomic nerve fibers were predominantly small caliber (Robinson-Papp et al., 2013), and it seemed reasonable that mitochondrial dysfunction and energetic failure in the distal axon (Robinson-Papp et al., 2013), as well as direct viral neurotoxicity, played a role in CAN development.

In view of the risk of fatal cardiorespiratory arrest, simple HRV tests could be useful in COVID-19 patients for the sudden cardiac death risk. The disturbance in the baroreflex mechanism causes cardiac conduction abnormalities and the detection of an autonomic disorder via the evaluation of HRV and BRS using non-invasive methods in patients with COVID-19 could alert clinicians to possible patient morbidity and mortality (Kocabas et al., 2018). Prospective study needs are warranted to determine the value of autonomic testing and regimes for the prevention and treatment of this complication of COVID-19 infection.

### Limitations

There are several limitations to the present study. This is a single-center, observational, cross-sectional study that has no insight into the sequence of events and advanced causatives in cardiovascular morbidity and mortality in COVID-19 patients. However, we consider this finding important for future prospective studies and a proper settlement of parameters analyzed in COVID-19 patients for adequate risk stratification and prediction regarding cardiovascular morbidity and mortality.

## Conclusion

As it has recently been accepted as a multisystem disorder due to its complex pathogenicity, ANS disorders in COVID-19 should be considered as the basis of various possible manifestations. Prominent sympathetic and parasympathetic dysfunction will be helpful in misperceived manifestations explanation, contributing to faster diagnosis and proper treatment of the patients.

## Data Availability Statement

The raw data supporting the conclusions of this article will be made available by the authors, without undue reservation.

## Ethics Statement

The studies involving human participants were reviewed and approved by the Ethics Committee of University Clinical Centre of the Republic of Srpska, Bosnia and Herzegovina, Number 01-5617. The patients/participants provided their written informed consent to participate in this study.

## Author Contributions

BM conceived of the presented idea. VD, AV, and PK carried out the collection of data. VD, AV, PK, and TK contributed to data collection. BM and MO verified the analytical methods. AD wrote the manuscript. BM, DB, and MO performed editing. All authors discussed the results and contributed to the final manuscript.

## Conflict of Interest

SJ was employed by the company Telekom Srbija a.d. The remaining authors declare that the research was conducted in the absence of any commercial or financial relationships that could be construed as a potential conflict of interest.

## Footnotes

1 https://omronhealthcare.com/covid-19/ (accessed March 19, 2021)

2 www.physiology.org.rs (accessed March 19, 2021)

3 https://eresearch.qmu.ac.uk/handle/20.500.12289/1 (accessed March 19, 2021)

4 https://www.ncbi.nlm.nih.gov/ (accessed March 19, 2021)

5 https://www.intechopen.com/books/subject/health-sciences (accessed March 19, 2021)

6 https://www.nemechekconsultativemedicine.com/vns-covid-19-white-paper/ (accessed March 19, 2021)
